# BDNF-induced nitric oxide signals in cultured rat hippocampal neurons: time course, mechanism of generation, and effect on neurotrophin secretion

**DOI:** 10.3389/fncel.2014.00323

**Published:** 2014-11-07

**Authors:** Richard Kolarow, Christoph R. W. Kuhlmann, Thomas Munsch, Christoph Zehendner, Tanja Brigadski, Heiko J. Luhmann, Volkmar Lessmann

**Affiliations:** ^1^Medical Faculty, Institute of Physiology, Otto-von-Guericke-UniversityMagdeburg, Germany; ^2^University Medical Center, Institute of Physiology, Johannes Gutenberg-University MainzMainz, Germany

**Keywords:** nitric oxide, BDNF, TrkB, PSD95, peptide secretion, neurotrophins, synaptic plasticity

## Abstract

BDNF and nitric oxide signaling both contribute to plasticity at glutamatergic synapses. However, the role of combined signaling of both pathways at the same synapse is largely unknown. Using NO imaging with diaminofluoresceine in cultured hippocampal neurons we analyzed the time course of neurotrophin-induced NO signals. Application of exogenous BDNF, NT-4, and NT-3 (but not NGF) induced NO signals in the soma and in proximal dendrites of hippocampal neurons that were sensitive to NO synthase activity, TrkB signaling, and intracellular calcium elevation. The effect of NO signaling on neurotrophin secretion was analyzed in BDNF-GFP, and NT-3-GFP transfected hippocampal neurons. Exogenous application of the NO donor sodium-nitroprusside markedly inhibited neurotrophin secretion. However, endogenously generated NO in response to depolarization and neurotrophin stimulation, both did not result in a negative feedback on neurotrophin secretion. These results suggest that a negative feedback of NO signaling on synaptic secretion of neurotrophins operates only at high intracellular levels of nitric oxide that are under physiological conditions not reached by depolarization or BDNF signaling.

## Introduction

Activity-dependent synaptic plasticity, and especially long-term potentiation (LTP), is considered as the cellular correlate of learning and memory processes in the mammalian brain. Extracellular synaptic messengers, like nitric oxide (NO), and brain-derived neurotrophic factor (BDNF) are important intercellular signaling molecules mediating some forms of LTP (Hawkins et al., 1998; reviewed in Hopper and Garthwaite, 2006; Gottmann et al., 2009; Edelmann et al., 2014). Both NO and BDNF are discussed as potential retrograde messengers of LTP in the CA1 region of the hippocampus (see e.g., Hawkins et al., 1998; Lessmann et al., 2011) and in various cortical synaptic circuits (Hardingham and Fox, 2006). Possible reciprocal interactions between NO and BDNF signaling pathways at the synaptic level are, however, not well understood.

The importance of NO in the induction and expression of LTP is supported by numerous studies. Inhibition of NO generation or genetic deletion of NO synthases (NOS) leads to reduced LTP in CA1 of the hippocampus (Schuman and Madison, 1991; Wilson et al., 1997) and the cerebral cortex (Haul et al., 1999; Volgushev et al., 2000). Endothelial as well as neuronal NOS (eNOS, nNOS) can contribute to hippocampal LTP. However, the degree to which eNOS and nNOS contribute to hippocampal LTP is controversial and is critically influenced by recording and stimulation conditions (compare e.g., Son et al., 1996; Wilson et al., 1997). Likewise, also the involvement of NO downstream signaling [e.g., guanylate cyclase, cGMP, protein kinase G (PKG)] in certain types of LTP relies on the specific experimental conditions applied (Schuman and Madison, 1994; Selig et al., 1996; Son et al., 1998; Bon and Garthwaite, 2003; Neitz et al., 2014). In any case, proper development of NO dependent hippocampal LTP seems to require phasic and tonic components of NO generation (Hopper and Garthwaite, 2006). NOS dependent LTP can be observed especially under conditions of weak synaptic stimulation, whereas more robust high frequency stimulation can evoke NOS independent LTP (O'Dell et al., 1994; Wilson et al., 1997). In hippocampal slices, simultaneous application of exogenous NO donors during enhanced synaptic basal stimulation with > 0.1 Hz evokes potentiation (Arancio et al., 1996; Zhuo et al., 1999; Bon and Garthwaite, 2001), and facilitates LTP at otherwise suboptimal stimulation frequencies (Bon and Garthwaite, 2003), whereas application of NO alone does not evoke a stable potentiation. Several lines of evidence suggest that NO acts as a classical retrograde messenger, that is generated in the postsynaptic neuron upon appropriate NMDA receptor activation, and travels back to the presynaptic neuron to induce enhanced glutamate release (Hardingham et al., 2013).

The importance of BDNF in hippocampal and neocortical LTP is also well established. LTP in CA1 and CA3 of the hippocampus, in the visual cortex, and in the amygdala is decreased under conditions of reduced availability of BDNF (Korte et al., 1995; Patterson et al., 1996; Kang et al., 1997; Itami et al., 2003; Abidin et al., 2006; Meis et al., 2012; Schildt et al., 2013). BDNF is released at synaptic structures upon high-frequency electrical stimulation (Hartmann et al., 2001) or after firing of theta burst trains of action potentials (Kuczewski et al., 2008), and mediates pre–and postsynaptic changes of AMPA receptor-mediated responses, which might both contribute to the maintenance of LTP (Lessmann et al., 1994; Carmignoto et al., 1997; Lessmann and Heumann, 1998; Itami et al., 2003). Similar to the role of NO, the influence of BDNF on LTP in CA1 is critically dependent on details of the LTP stimulation protocol (Kang et al., 1997; Patterson et al., 2001; Zakharenko et al., 2003).

Given the conjunct importance of NO and BDNF in LTP, molecular cross talk between the respective signaling cascades is likely to exist. Previous data indeed suggest an interaction of both pathways. Upon the one hand, NO production can be stimulated by addition of BDNF (Koh et al., 1995; Samdani et al., 1997; Klöcker et al., 1998), on the other hand global release of BDNF from hippocampal slices is inhibited by the NO/cyclic GMP signaling pathway (Canossa et al., 2002). However, generation of NO in response to BDNF, and effects of NO on depolarization-induced synaptic BDNF secretion, have as yet remained elusive.

Our study now provides evidence, that BDNF induces generation of NO in hippocampal neurons (somata and dendrites) on a fast time scale. This NO elevation requires TrkB signaling and subsequent calcium elevation. We further show that excessive generation of NO in hippocampal cultures can reduce synaptic BDNF secretion. However, endogenously released BDNF is not able to increase intracellular NO levels sufficiently to exert a negative feedback on BDNF secretion. Thus, the NO-BDNF feedback loop does not seem to be activated by endogenous levels of BDNF and NO upon synaptic stimulation.

## Results

### Time course and localization of BDNF-induced NO signals in neurons

BDNF-induced generation of NO in cortical neurons has been reported previously using DAF imaging (Hwang et al., 2002; Nott et al., 2008) or quantification of L-arginine consumption (Riccio et al., 2006). To reveal the location and the time course of production of endogenous NO in response to exogenously added BDNF (100 ng/ml) in our hippocampal cells, microcultures of rat hippocampal neurons (15–18 DIV) were loaded with the NO indicator DAF and generation of NO was visualized with time-lapse confocal microscopy (see Materials and Methods).

**Figure 1:**
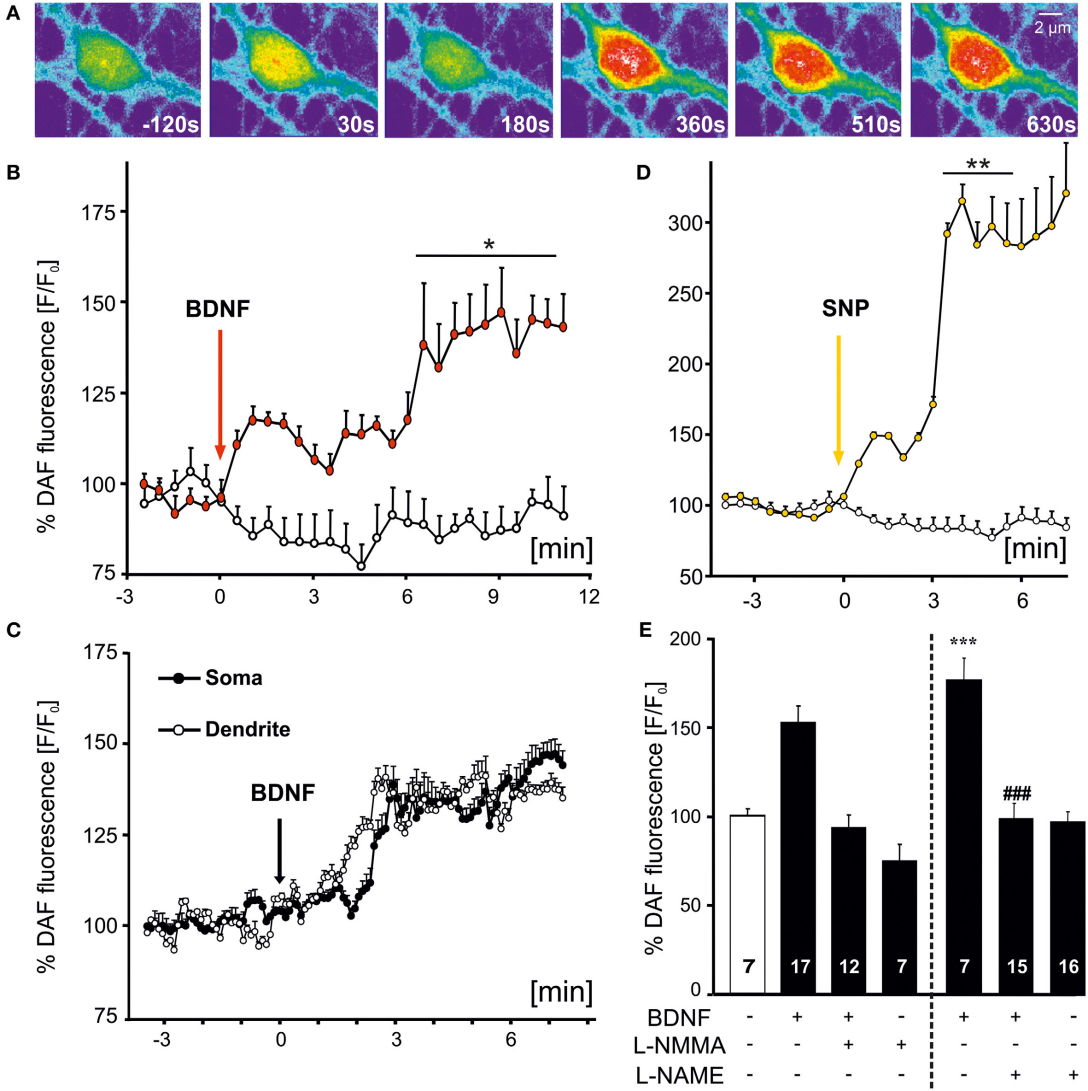
**Time course of BDNF-induced NO signals in hippocampal neurons**. Microcultures of rat hippocampal neurons (15–18 DIV) were loaded with the fluorescent NO indicator DAF, and changes in fluorescence intensity of DAF were monitored using time-lapse confocal microscopy. **(A)** Images of BDNF (100 ng/ml, bath application starting at 0 s)-induced NO signal in a single hippocampal neuron at time points as indicated. Note the increase of NO in the soma and proximal dendrites. **(B)** Average (*n* = 9 cells from 3 experiments) NO increase induced by bath applied BDNF (100 ng/ml) vs. negative control (continuously superfused with HBS). Vertical arrow indicates time point of drug application, ^*^p < 0.01. **(C)** Averaged parallel NO increase in soma vs. dendrites in the same individual cells (*n* = 5; different cells than shown in **B**). **(D)** Average (*n* = 5 cells) NO increase induced by SNP (100 μM), used as positive control, ^**^p < 0.001 vs. negative control. **(E)** Mean BDNF-induced DAF fluorescence intensity 10 min after start of stimulation. Drug application (100 ng/ml BDNF, 300 μM L-NMMA, 10 μM L-NAME) as indicated. Note the complete inhibition of BDNF-induced NO signals in the presence of NOS inhibitors. ^***^p < 0.001 vs. control; ### *p* < 0.001 BDNF + L–NAME vs. BDNF. Errors bars represent s.e.m.

Bath application of BDNF-induced an increase of NO that was evident by the fluorescence increase of DAF (Figures 1A,B). The increase in DAF fluorescence was detected in all (*n* = 24 cells from 9 experiments) hippocampal neuronal cultures investigated, but was not observed in co-cultured astrocytes in the same fields of view. The NO increase became visible within the first 30 s of BDNF application, further increasing in amplitude upon sustained BDNF stimulation (Figure 1B). On average, somatic BDNF-induced fluorescence increased roughly 1.5 fold compared to control levels (at 10 min, control: 0.88 ± 0.04, *n* = 19 hippocampal neurons from 3 experiments; BDNF: 1.41 ± 0.09, *n* = 9 cells from 3 experiments). Next we aimed at analysing the time course of dendritic and somatic NO levels in the same neurons. In this series of experiments we selected 5 cells in the same field of view with non-fluctuating DAF signals before and after application of BDNF. This experiment revealed BDNF-induced NO generation concomitantly in soma and dendrites of the identical hippocampal neurons and the increase was of similar amplitude in both compartments (Figure 1C). As a positive control for the time course and amplitude of NO signaling in hippocampal neurons, we applied the NO donor sodium nitroprusside (SNP, 100 μM), leading to nearly threefold increase of baseline fluorescence values (at 5 min, control: 0.70 ± 0.18%, *n* = 19; SNP: 2.85 ± 0.28%, *n* = 9; Figure 1D). Inhibition of NOS by preincubating neurons with L-NMMA (300 μM, 30 min) completely blocked the BDNF-induced elevation of intracellular NO levels (Figure 1E). This confirms the specificity of the assay for the detection of intracellular NO increase (at 10 min, control: 100.7 ± 3.8%, *n* = 7; BDNF: 153.8 ± 9.4%, *n* = 17; BDNF plus L-NMMA: 94.1 ± 6.9%, *n* = 12; L-NMMA: 75.1 ± 9.1%, *n* = 7). A similar degree of inhibition of the BDNF-induced NO signal was observed when cells were preincubated with the unselective NO synthase inhibitor L–NAME (10 μM) (control: 100.1 ± 4.5%, *n* = 7; BDNF: 178.6 ± 12.1%, *n* = 7, *p* < 0.001 vs. control; BDNF plus L-NAME: 100.4 ± 8.1%, *n* = 15, *p* < 0.001 vs. BDNF; L-NAME: 99.9 ± 5.3%, *n* = 16).

### Pharmacological profile of BDNF-induced NO generation

**Figure 2:**
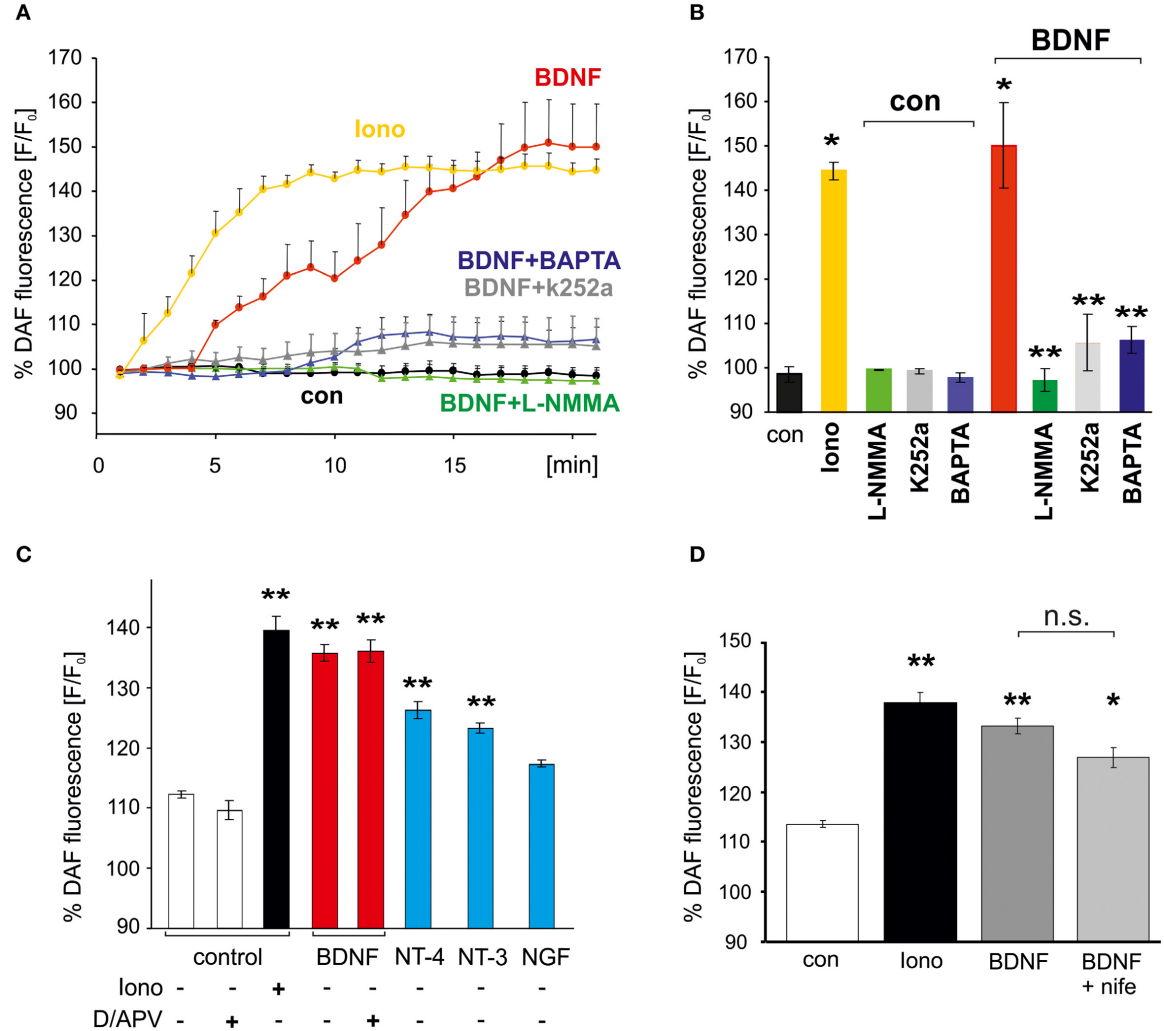
**Activation of Trk receptors and intracellular Ca^2+^ elevation are required for BDNF-induced NO generation**. Cultured hippocampal neurons (15–18 DIV) were loaded with the NO indicator DAF, and DAF fluorescence of wells was monitored with a plate reader. **(A)** Time course of average fluorescence intensities (*n* = 4) is shown for drug treatments (at 1 min, 1 μM Ionomycin, 300 μM L-NMMA) as indicated. **(B)** Average DAF fluorescence 20 min after treatments as indicated. Ionomycin served as positive control. ^*^p < 0.01 compared with negative control, ^**^p < 0.001 compared with BDNF positive control. Note the almost complete block of BDNF-induced NO signals by BAPTA (10 μM) and k252a (200 nM). Error bars represent s.e.m. **(C)** NO signals induced by BDNF, NGF, NT-3, NT-4, respectively (100 ng/ml, each; average DAF fluorescence 20 min after treatment as indicated). Note the independence of BDNF-induced NO signaling from inhibition of glutamatergic transmission (D/APV = 10 μM DNQX, 50 μM APV). **(D)** Inhibition of L–VGCCs with nifedipine (10 μM, average DAF fluorescence after 20 min treatment) did not affect BDNF-induced NO signals. ^*^p < 0.05, ^**^p < 0.01 compared with negative control. Error bars represents s.e.m.

To facilitate quantitative analysis of the signaling cascades involved in BDNF-induced generation of NO, DAF fluorescence of hippocampal neurons was also determined with a plate reader assay (see Materials and Methods). Similar to the results obtained with the confocal microscope, incubation of the cells with BDNF revealed an NO increase to roughly 150% of control values (at 20 min, BDNF: 150 ± 9.6% of control values, *p* < 0.01 vs. control; *n* = 4 independent experiments, Figures 2A,B). The increase in DAF fluorescence in BDNF treated cells was comparable to the increase following application of 1 μM of the calcium ionophore ionomycin (at 20 min, Ionomycin: 144.3 ± 2.1% of control values, *p* < 0.01 vs. control), which served as a positive control in subsequent experiments. The BDNF-induced NO elevation was significantly reduced (compare Figures 2A,B) by the NO synthase inhibitor L–NMMA (300 μM; BDNF + L–NMMA: 97.2 ± 2.5%; L–NMMA: 99.2 ± 0.1% of control values, *p* < 0.001 vs. BDNF + L–NMMA), the TrkB receptor kinase inhibitor k252a (200 nM, 30 min preincubation; BDNF + k252a: 105.6 ± 6.3%; k252a: 99.2 ± 0.6% of control values, *p* < 0.001 vs. BDNF + k252a; *n* = 4 independent experiments for each treatment, Figure 2B), and by inhibiting elevation of intracellular Ca^2+^ by preincubation with the membrane permeable Ca^2+^ chelator BAPTA (10 mM; BDNF + BAPTA: 106.3 ± 2.9%; BAPTA: 97.7 ± 1.0% of control values, *p* < 0.001 vs. BDNF + BAPTA, Figure 2B). These data indicate, that the BDNF-induced NO increase is mediated via activation of TrkB receptors, and subsequent intracellular Ca^2+^ elevation, eventually leading to NO synthase activation.

The neurotrophins NT-3 and NT-4 (100 ng/ml each) also induced a clear, albeit smaller increase of NO (Figure 2C), whereas NGF did not lead to a significant NO elevation. These data indicate that TrkB and TrkC activation can initiate NO signaling in our neurons, with highest efficiency after stimulation of TrkB with BDNF.

BDNF application is well known to increase neuronal activity by enhancing synaptic strength (reviewed e.g., in Gottmann et al., 2009). Due to the relatively slow onset of the BDNF-induced NO elevation we therefore further investigated whether the observed NO increase was secondary to enhanced excitatory synaptic activity in response to BDNF. However, when BDNF was applied under conditions of complete blockade of glutamatergic synaptic transmission (using 10 μM DNQX, 50 μM APV) no significant inhibition of the BDNF-induced NO increase was observed (Figure 2C). We thus conclude that BDNF-induced NO elevation in hippocampal neurons occurs independent from enhanced glutamatergic synaptic network activity. Since BDNF-induced NO elevation was not significantly inhibited in the presence of the L-type voltage gated calcium channel (VGCC) inhibitor nifedipine (100 μM; Figure 2D) we conclude that calcium influx via L-type VGCC can not account for the calcium increase that triggers BDNF-induced NO signals.

### NO-dependent modulation of postsynaptic release of neurotrophins

**Figure 3:**
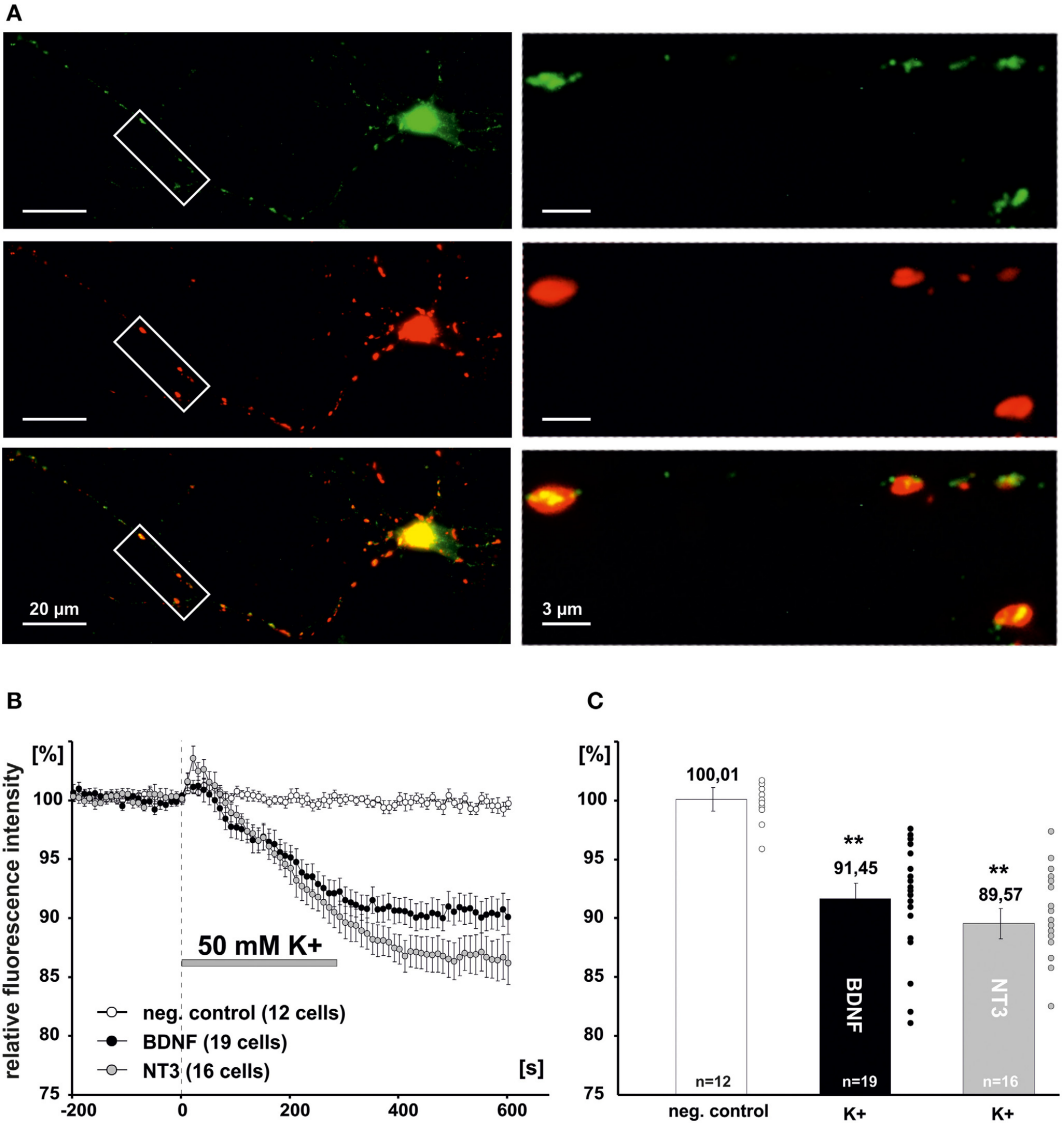
**Measurement of synaptic release of neurotrophins**. **(A)** Hippocampal neurons were co-transfected at 8-DIV with BDNF-GFP and PSD95-DsRed. Co-localization of both proteins was monitored at 10–11 DIV. Boxed areas on the left are shown at higher magnification on the right. Postsynaptic vesicle clusters of BDNF-GFP (green) were identified by co-localization with PSD95-DsRed (Red). **(B)** Time course of postsynaptic BDNF-GFP and NT-3-GFP release in response to depolarization with elevated K^+^ (50 mM, 300s). **(C)** Residual fluorescence of postsynaptic BDNF-GFP and NT-3-GFP after 300 s depolarization. Experiments were performed in the presence of 10 μM DNQX, 200 μM D,L-APV, and 10 μM gabazine, to avoid secondary effects via transmitter secretion. ^**^p vs control < 0.0001. Error bars represent s.e.m.

Since previous reports suggested a negative effect of NO signaling on global BDNF secretion from hippocampal slices (Canossa et al., 2002), we asked whether a similar regulatory effect could be observed for postsynaptic secretion of BDNF at glutamatergic synapses. Cultured hippocampal neurons were cotransfected at 8–9 DIV with PSD-95-DsRed (a postsynaptic marker of glutamatergic synapses) and BDNF-GFP or NT-3-GFP, respectively. Two days after transfection, postsynaptic clusters of NT containing secretory granules were identified by colocalization of the NT-GFP signal with PSD-95-DsRed (Figure 3A). Secretion of BDNF and NT-3 from postsynaptic sites was induced by application of high K^+^ solution (50 mM, 5 min) and measured by analyzing the decrease of intracellular GFP fluorescence intensity using time-lapse video microscopy, as described previously (Brigadski et al., 2005; see Materials and Methods). Inhibitors of synaptic transmission were added (100 μM APV, 10 μM DNQX, 50 μM Gabazine) to exclude any indirect effects of glutamatergic and GABAergic synaptic activity on the secretion process. On average, this depolarizing stimulation led to a release of about 10% of stored NT content of the secretory vesicles (i.e., remaining intracellular fluorescence for BDNF-GFP: 91.5 ± 1.3%, *n* = 19 cells; NT-3: 89.6 ± 1.3%, *n* = 16 cells; 50 mM K^+^ vs. negative control significantly different with *p* < 0.0001; Figures 3B,C).

**Figure 4:**
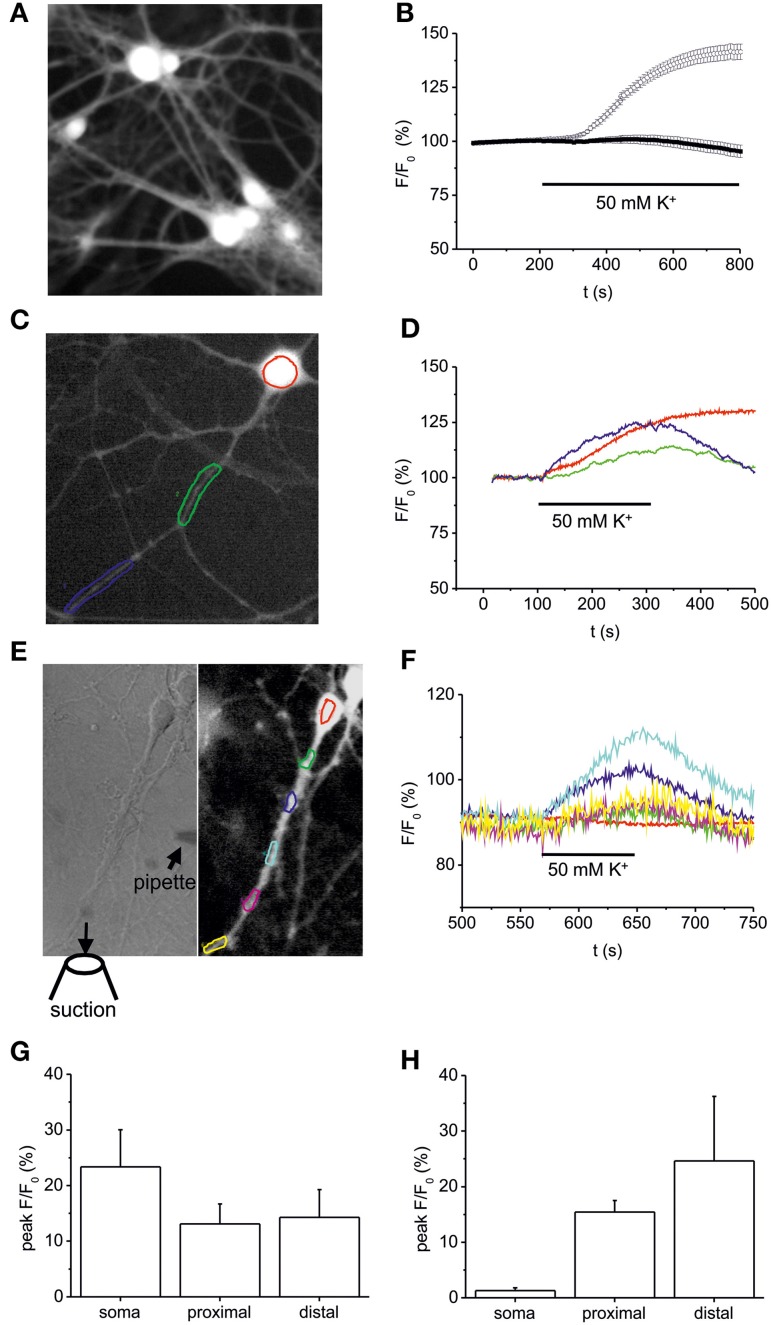
**Somatic vs. dendritic NO signals induced by 50 mM K^+^-induced stimulation**. Hippocampal neurons (10–12 DIV) were loaded with the fluorescent NO indicator DAF-FM, and changes in DAF fluorescence intensity were monitored using time-lapse video microscopy. **(A)** Typical example of DAF-FM loaded hippocampal neurons following stimulation with 50 mM K^+^ used for analysis of fluorescence intensity in the soma as shown in **(B)**. **(B)** time course of DAF fluorescence upon stimulation with 50 mM K^+^ starting at 200 s. Average DAF fluorescence intensity in hippocampal neurons exposed to 50 mM K^+^ solution under control conditions (*n* = 25 cells; open circles) and after pre-incubation (10 min) in the presence of 300 μM L-NMMA (*n* = 32; black squares). **(C,D)** Typical DAF-FM loaded hippocampal neuron with color coded somatic and dendritic regions analyzed in **(D)**. Note the simultaneous increase in DAF fluorescence in somatic (red), proximal (green), and distal dendrites (blue). **(E)** Subcellular resolution of DAF-FM signals following local pressure-application of high K^+^ (50 mM). High K^+^ solution was applied via a small pipette (left hand image) directed toward the dendrite of a hippocampal neuron. The suction pipette was positioned such that direct depolarization of the soma was avoided. **(F)** Normalized DAF fluorescence of color coded regions marked in E indicates simultaneous NO-generation in dendritic compartments, whereas no increase was detected in the cell soma. **(G)** Average peak increase in DAF fluorescence in 5 independent experiments as shown in **(C,D)**. **(H)** Average peak increase in DAF fluorescence in 5 independent experiments as shown in **(E,F)**. Data represent means ± s.e.m.

To elucidate whether the high K^+^-induced depolarization used to provoke NT secretion rises intracellular NO levels we performed DAF-FM imaging (Figure 4A). Bath application of 50 mM K^+^ (10 min) induced an increase of intracellular NO in somata of hippocampal neurons to 141.5 ± 3.6% (at 500 s following stimulation; *n* = 25) as compared to control (99.5 ± 1.3%; Figure 4B). This increase was similar in amplitude to BDNF (100 ng/ml)-induced NO elevation (compare Figures 1, 2), and was abolished when high K^+^ solution was applied in the presence of L-NMMA (300 μM; 95.3 ± 2.9%, *n* = 32; Figure 4B). Further analysis of short (100–200 s) pulses of high K^+^ bath application revealed a concomitant elevation of NO (all values at 300 s following stimulation) in somata (23.4 ± 6,7%), proximal (13.1 ± 3.6%), and distal dendrites (14.3 ± 5%) without delay between soma and processes (*n* = 7, Figures 4C,D,G), suggesting that diffusion of NO from the soma into dendrites of hippocampal neurons does not account for dendritic NO signals. When using focal application of depolarizing 50 mM K^+^ solution via a small diameter pipette (Figure 4E), NO signals could be elicited selectively in dendrites, demonstrating that dendritic NO elevation can occur independent from somatic NO elevation (*n* = 6, Figure 4F). On average, local application of high K^+^ to dendrites caused no significant change in NO levels (1.3 ± 0.5%) in somata, but a simultaneous increase to 15.4 ± 2.1% in proximal and 24.6 ± 11.6% in distal dendrites (*n* = 6 cells, Figure 4H).

**Figure 5:**
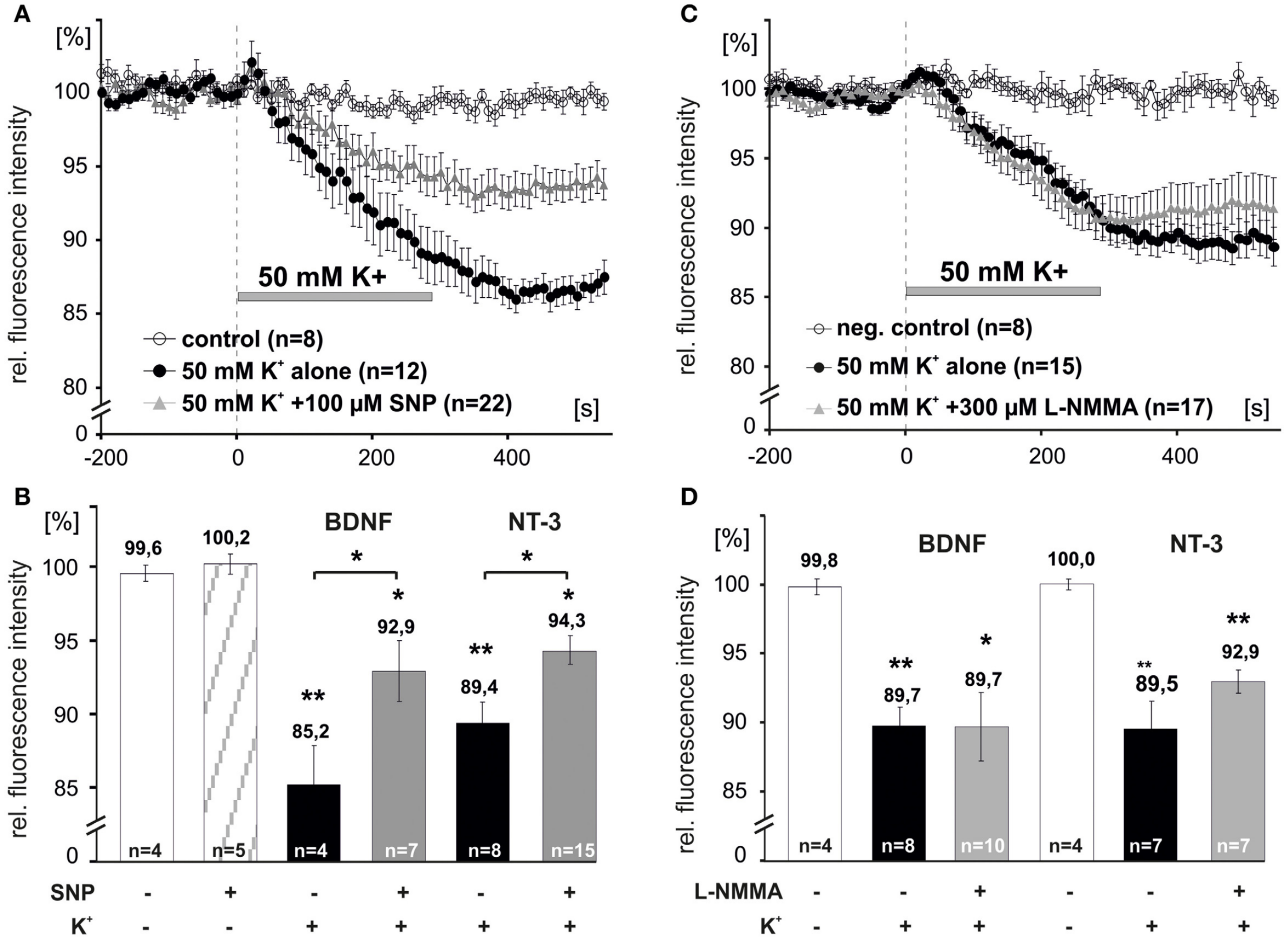
**NO dependent modulation of synaptic release of BDNF and NT-3**. Hippocampal neurons were transfected with BDNF-GFP (BDNF) or NT-3-GFP (NT-3) and monitored for neurotrophin secretion. **(A,C)** Averaged depolarization-induced (50 mM K^+^) release of NTs vs. negative control. (**B,D)** Mean residual fluorescence 300s after stimulation. **(A,B)** Preincubation with the NO donor SNP (100 μM, 5 min) reduced depolarization-induced secretion of neurotrophins. **(C,D)** Preincubation and subsequent superfusion with the NOS inhibitor L–NMMA (300 μM, 5 min) during depolarization did not change the amount of neurotrophins that was released. Experiments were performed in the presence of 10 μM DNQX, 200 μM D,L-APV, and 10 μM gabazine, to avoid secondary effects via transmitter secretion. Negative control cells were superfused with HBS throughout. ^*^p vs. control < 0.05, ^**^p vs control < 0.005. Error bars represent s.e.m.

We next asked, whether elevation of intracellular levels of NO can directly initiate the release of NTs. Acute application of the NO donor sodium nitroprusside (SNP, 100 μM, 10 min) alone did not induce release of either BDNF or NT-3 (remaining intracellular fluorescence for acute SNP: 100.2 ± 0.7%, *n* = 5; Figure 5B). However, 5 min preincubation with SNP (100 μM,) significantly reduced depolarization-induced NT secretion (BDNF + SNP: 92.9 ± 2.1%, *n* = 7; BDNF control: 85.2 ± 2.7%, *n* = 4; NT-3 + SNP: 94.3 ± 1.0%, *n* = 15; NT-3 control: 89.4 ± 1.4%, *n* = 8, *p* < 0.05; Figures 5 A,B). We further tested whether inhibition of NOS activity by preincubation with L–NMMA (300 μM, 15 min; a concentration effectively interfering with NO generation by exogenous BDNF and by high K^+^, respectively; compare Figures 1, 4) would interfere with depolarization-induced synaptic neurotrophin secretion. Interestingly, this paradigm failed to reveal a significant reduction of either BDNF or NT-3 secretion (BDNF + L-NMMA, 89.7 ± 2.5%, *n* = 10; BDNF control, 89.7 ± 1.4%, *n* = 8; NT-3 + L-NMMA, 92.9 ± 0.8%, *n* = 7; NT-3 control, 89.5 ± 2.0%, *n* = 7, not significantly different with *p* > 0.05; Figures 5C,D). This result suggests that the robust depolarization of neurons that is sufficient to release neurotrophins, did not elevate intracellular NO up to a level that shows a negative feedback on neurotrophin secretion.

## Discussion

Here we show that BDNF induces NO generation in the soma and dendrites of hippocampal neurons in a calcium dependent manner and independently from glutamatergic synaptic activity. Our time resolved imaging demonstrates that the BDNF-induced production of NO occurs within 1–2 min in dendrites and in the soma of hippocampal neurons. This NO generation occurred independent of the enhancing effect of BDNF on glutamatergic synaptic activity, but appeared to be a direct consequence of BDNF/TrkB-induced calcium elevation in the cells. Reciprocally, strongly enhanced levels of NO inhibit postsynaptic secretion of BDNF in hippocampal neurons. However, baseline levels of NO, and levels of NO that were reached in response to high K^+^-induced depolarization and subsequent synaptic secretion of BDNF or NT-3 were below threshold to modulate neurotrophin secretion.

### Activation of NOS downstream of BDNF/TrkB signaling

The finding that BDNF can directly trigger NO production in hippocampal neurons is in agreement with previous data showing BDNF-induced NO signals in the somata of cultured cortical neurons (Hwang et al., 2002; Nott et al., 2008), and as shown indirectly by L-arginine consumption in cortical cultures (Riccio et al., 2006). Our data now reveal the time course of NO production in hippocampal neurons. BDNF-induced NO elevation can be observed in the soma, as well as in the dendrites within 1–2 min following bath application of high concentrations of BDNF (100 ng/ml). Similarly, high K^+^-induced depolarization of the neurons also elicited an intracellular NO increase that was similar in amplitude and showed a comparable time course as BDNF-induced NO elevation (compare Figures 1, 4). This high K^+^-induced depolarization was further successful in eliciting NT secretion from our neurons (Figures 3, 5), and is also able to initiate secretion of *endogenous* BDNF in hippocampal neurons (Kuczewski et al., 2008). Although in our release experiments (i) high K^+^, (ii) secreted endogenous BDNF, and (iii) secreted BDNF-GFP, all have the capacity to elevate intracellular NO levels, this NO elevation was obviously insufficient to reach the threshold for NO dependent modulation of neurotrophin secretion, that could be observed in the presence of the NO donor SNP (compare below).

### Mechanism of BDNF-induced NO elevation

Interestingly, generation of NO in response to BDNF was neither dependent on AMPA receptor mediated synaptic network activity, nor on the activation of NMDA receptors, or on the activity of L-type VGCCs, albeit critically relying on intracellular calcium elevation (compare Figure 2). Thus, our data suggest that the Ca^2+^ elevation responsible for NOS activation relies mainly on calcium release from intracellular stores, as is expected for BDNF/TrkB signaling (see e.g., Berninger et al., 1993; Marsh and Palfrey, 1996). This interpretation is also in line with the slightly delayed onset of NO generation in response to BDNF (compare Figures 1, 2) which is typical for a second messenger cascade-induced calcium release from internal stores. It remains to be shown whether BDNF-induced activation of TRPC3 calcium channels (compare Amaral and Pozzo-Miller, 2007; Nakata and Nakamura, 2007) also contributes to the BDNF-induced NO synthesis.

NT-3 or NT-4 (but not NGF) also induced NO elevation comparable in amplitude to BDNF-induced NO signals in our cells. Hence, our data suggest that TrkB and TrkC (but not TrkA), signaling are both effective in NO production in our cells. The absence of TrkA-induced NO elevation might be explained by the lower abundance of TrkA (compared to TrkB and TrkC) in cultured hippocampal neurons (Ip et al., 1993). Given the inhibition of BDNF-induced NO signaling by the Trk kinase inhibitor k252a, involvement of TrkB signaling in generation of NO seems warranted.

### Modulation of neurotrophin secretion by NO signaling

Our observation that exogenous supply of NO inhibits the secretion of neurotrophins is in line with previous observations (Canossa et al., 2002). These authors reported that exogenous application of activators of the cGMP/PKG signaling pathway decreased basal/spontaneous BDNF secretion similar to NO donors (Canossa et al., 2002). We now investigated the effect of NO signaling on depolarization-induced (evoked) secretion of neurotrophins at glutamatergic synapses, and found that raising NO levels artificially by a nitric oxide donor (SNP), showed a similar inhibition of secretion of BDNF and NT-3 in our assay.

Since the depolarizing stimulus induced by application of 50 mM K^+^ can by itself stimulate NO generation (compare Figure 4), the most sensitive test for the contribution of endogenously produced NO to modulation of neurotrophin secretion is application of a NOS inhibitor (L-NMMA, 300 μM) during high K^+^ depolarization. Importantly, L–NMMA had no effect on depolarization-induced secretion of BDNF or NT-3 (compare Figure 5), although the inhibitor was effective in blocking BDNF as well as high K^+^-induced generation of NO (Figures 1, 4). Taken together, these data indicate that the endogenous NO levels that are reached even by the robust depolarization with 50 mM external K^+^ are not sufficient to inhibit neurotrophin secretion.

Our finding that inhibition of endogenous baseline NO production did not affect neurotrophin secretion is at variance with previously published results (Canossa et al., 2002). It remains to be determined whether these contrasting results originate from differences in experimental procedures, differences in the type of secretion that was investigated (i.e., global basal secretion from somata and neurites was investigated by Canossa and coworkers, whereas depolarization-induced synaptic secretion was investigated in our present study), or differences in the baseline level of excitatory synaptic transmission in these two neuronal culture systems.

### Crosstalk between BDNF and NO signaling

The existence of an interplay between NO and BDNF signaling has been shown previously for a number of biological effects other than neurotrophin secretion. Exogenously applied BDNF induces expression of nNOS and basal expression of NOS is controlled by the endogenous levels of BDNF (Samdani et al., 1997; Xiong et al., 1999). In addition, NO synthesis can be stimulated by exogenous BDNF (Klöcker et al., 1998), and a positive feedback of BDNF on NO generation can enhance NO-mediated neuronal necrosis (Koh et al., 1995; Samdani et al., 1997). Finally, BDNF and NO can promote synergistically neuronal fiber outgrowth (Ernst et al., 2000). Conversely, NO - apart from negatively regulating BDNF secretion - exerts mainly a negative regulation of BDNF signaling. Endogenous NO, presumably released by application of BDNF, inhibits the neuroprotective effect of BDNF after axotomy of retinal ganglion cells (Klöcker et al., 1998), and NO inhibits BDNF synthesis in neocortical neurons (Xiong et al., 1999). In contrast, *in vivo* studies showed NO-induced increased BDNF production, which seems to be involved in the formation of spatial memory (Mizuno et al., 2000). Nevertheless, all these studies investigated cross talk between both signaling pathways on the time scale of hours, thus not excluding intermediate secondary effects for this reciprocal modulation of both signaling cascades. In our study, using BDNF and NO measurements at higher spatial and time resolution, and taking into account that we could exclude intermediate synaptic network effects in our measurements, our data indicate a direct connection of BDNF and NO signaling at the cellular level.

At the molecular level, there is evidence for a direct positive interaction between NO and BDNF signaling pathways, as shown e.g., by the direct activation of TrkB receptors through NO-induced formation of peroxynitrite (Yuen et al., 2000). Interestingly, the small G-protein p21ras, which is activated downstream of TrkB signaling, is a target for S-nitrosylation, giving potentially rise to NO-induced activation of MAP kinases (Lander et al., 1996).

Importantly, several studies showed a postsynaptic localization of both nNOS and TrkB receptors in the postsynaptic density of glutamatergic synapses of the hippocampus, and nNOS, but not eNOS, is a component of the NMDA receptor complex (Husi et al., 2000). In CA1/CA2 of rat hippocampus, NR2A- or NR2B- containing NMDARs associate with PSD-95, and NR2A coimmunoprecipitates with nNOS (Al-Hallaq et al., 2007). PSD-95 is necessary for the association of the nNOS/NMDAR complex, where it associates with both proteins through the second PDZ domain (Christopherson et al., 1999; Cui et al., 2007). Also other components of the NO/cGMP/PKG signaling pathway, like e.g., the NO-sensitive soluble guanylyl cyclase (sGC) are localized in part in postsynaptic densities (Burette et al., 2002). Interestingly, PKG which is a major effector of the NO/sGC/cGMP pathway has been shown previously in studies in non-neuronal cells, to be permissive for regulated exocytosis. (Li et al., 2004; Nanamori et al., 2007), suggesting a crosstalk between NO signaling and peptide secretion also outside the central nervous system.

Taken together, these studies suggest a direct interaction of BDNF and NO signaling pathways in postsynaptic structures, emphasizing that they can act in concert on synaptic plasticity.

## Materials and methods

### Cell culture

Dissociated postnatal rat hippocampal microcultures were prepared as described previously (Lessmann and Heumann, 1998; Brigadski et al., 2005). Primary postnatal rat (P0-P2) neocortical astrocytes were isolated and cultured for 2–4 weeks in DMEM medium, containing 10% FCS until expanded to confluence. Astrocytes were passaged and seeded on glass cover slips at a density of 80.000 cells per 3.5 cm dish in DMEM/10% FCS, to yield astrocyte islands of 100–300 μm in diameter after 7–14 days *in vitro* (DIV). Five μM ARAC was added 5 days after seeding of astrocytes to avoid further growth of astrocyte islands. For single-cell time-lapse measurements, dissociated postnatal rat (P0–P2) hippocampal neurons were plated in DMEM/10% FCS at a density of 1–10 neurons per astrocyte island onto the astrocyte cover slips.

For measurement of NO signals with a plate reader, dissociated rat hippocampal neurons were plated in DMEM/10% FCS at a density of 30–60.000 neurons per well in poly-ornithine coated 96 wellplates. After 20 h the plating medium was exchanged to serum-free medium (Neurobasal with 2% B27 supplement, Invitrogen).

### Transfection

Rat hippocampal microcultures were transfected with the respective expression plasmids (3 μg DNA per 3.5 cm dish) at 8–9 DIV, using the Ca^2+^ phosphate precipitation method as described previously (Haubensak et al., 1998). During incubation (1.5–3 h), 10 μM DNQX and 100 μM D,L-APV were added to reduce excitotoxicity. After one to two hours the transfection medium was replaced, cultures were washed 3 times with warmed HBS, and the cultures were kept in neuron-conditioned Neurobasal/B27 medium, thereafter. The cells were used for time lapse imaging experiments 1–3 days after transfection.

### Cotransfection with synaptic marker proteins

The construction and use of the PSD95-DsRed expression plasmid was described previously (Brigadski et al., 2005). Intact synaptic targeting of PSD95-DsRed was confirmed by colocalization of PSD95-DsRed with the green fluorescent activity-dependent label of active synapses, FM 1–43 (data not shown). To identify postsynaptic structures, in BDNF-GFP and NT-3-GFP expressing hippocampal neurons, cells were cotransfected with the respective NT-GFP construct together with PSD95-DsRed (DNA ratio GFP:DsRed, 1:1, 3 μg per dish overall).

### Epifluorescence imaging

Visualization of epifluorescence signals was performed as described previously (Hartmann et al., 2001). Briefly, cover slips with transfected cells were transferred into Petriperm dishes (Vivascience) with folio bottom and inspected with an inverted microscope (Olympus IX70) using 40 × (N.A.: 1.0) and 100 × (N.A.: 1.35) oil immersion objectives. Image capture was performed using a cooled CCD camera (Sensys 1401E, Photometrics), controlled by MetaVue software (Molecular Devices). Exposure times were chosen such that saturation was avoided. Processing of images was performed by MetaVue and Adobe Photoshop software without compromising the evident primary image information. However, in the figures, fluorescence in the soma and the proximal dendrites is often enhanced close to saturation in order to make single vesicles in *distal* neurites also clearly visible.

Some of the experiments were performed using an upright microscope (Zeiss, Axioscope FS) equipped with a 40 × objective (Olympus, LumPlanFI, N.A. 0.8 w). DAF-FM fluorescence was exited at 485 nm with a VisiChrome high speed monochromator (Visitron, Puchheim, Germany). Fluorescence images were taken with a Photometrics CoolSnap HQ2 CCD camera (Roper Scientific) at 0.1 Hz after passing a 510 nm long pass filter. Image acquisition and analysis was performed using Metafluor software (Molecular Devices).

### Confocal imaging

Analysis of the subcellular distribution of DAF fluorescence signals was performed with a Nipkow spinning disk confocal system (Visitech, England) attached to a conventional fluorescence microscope (Olympus BX51 WI) equipped with a high aperture water immersion objective (60 ×, n.A. 0.9). Full frame image capture was performed with a cooled CCD camera (CoolSnap HQ, Roper Scientific). Green and red fluorescence was excited with the 488 nm and the 568 nm lines, respectively, of a Kr/Ar laser (Laser Physics, U.S.). Image capture and analysis was performed using Metamorph software (Molecular Devices).

### Measurement of intracellular NO levels

Rat hippocampal neurons were incubated 30 min in Neurobasal medium at 37°C with diaminofluoresceine (DAF, 100 μM). The cells were washed once with HEPES buffered saline (HBS: 100 mM NaCl, 4 mM KCl, 20 mM HEPES, 0.9 mM Na_2_HPO_4_, 2 mM CaCl, 1 mM MgCl_2_, 10 mM glucose, 0.01 mM glycine, pH 7.4). Then the cells were preincubated 15–20 min at 37°C with inhibitors and used subsequently for single cell time-lapse recording of DAF fluorescence with a Nipkow spinning disk confocal microscope (excitation at 488 nm; emission apps 500–550 nm).

For some of the experiments cultured hippocampal neurons (10–12 DIV) were incubated with DAF-FM (10 μM) for 60 min in HBS buffer at 35°C. After dye loading, coverslips were transferred to the experimental chamber and washed for 15 min with fresh HBS. During experiments, cells were constantly superfused with fresh HBS.

For analyzing DAF fluorescence in 96 well plates, measurements were performed with an Infinite™ 200 plate reader at 37°C (Tecan, Germany, excitation at 485 nm). The cells were stimulated 6 min after the beginning of measurement. For each condition, the data were normalized to the mean value obtained during the preincubation time. Measurements not showing a stable baseline in the absence of stimulation during the first 6 min of recording, were excluded from analysis.

### Measurement of neurotrophin release

Real time imaging of synaptic secretion of GFP-tagged NTs was performed by analyzing the decrease of intracellular GFP fluorescence, as described previously (Brigadski et al., 2005). Cells with synaptically localized NT-GFP were superfused locally with HBS throughout the experiment, and images were captured at 10 s intervals. After 5 min control period, cells were depolarized (5 min) by superfusing 50 mM K^+^ containing (replacing an equal amount of Na^+^) HBS at RT. Regions with synaptic clusters of NT-GFP were analyzed. Background fluorescence levels of a void region in the same field of view was recorded in parallel and subtracted. Fluorescence decrease due to photobleaching was corrected for by extrapolating the fluorescence decrease during an initial 5 min control period over the whole recording time. Original fluorescence values at a given time point were normalized to the corresponding value of this extrapolated bleaching curve, thus providing a measure of the fluorescence decrease, which is due to release. This fluorescence decrease has been shown previously to reflect tetanus toxin and Ca^2+^ sensitive release of NTs (see Hartmann et al., 2001).

### Reagents

K252a and human recombinant neurotrophins were purchased from Alamone Labs (Jerusalem, Israel). BAPTA, DNQX, d,l-APV, gabazine, L–NMMA, L–NAME and sodium nitroprusside (SNP) were obtained from Sigma, DAF-2A and ionomycin were from Calbiochem. DAF-FM diacetate was purchased from Invitrogen.

### Conflict of interest statement

The Review Editor Thomas Mittmann declares that, despite sharing the same affiliation as the authors, the review process was handled objectively. The authors declare that the research was conducted in the absence of any commercial or financial relationships that could be construed as a potential conflict of interest.
